# 632. Characterization of Cytomegalovirus (CMV) Reactivation in Patients with Malignant and Non-Malignant Hematological Diseases at large Academic Center in Saudi Arabia

**DOI:** 10.1093/ofid/ofad500.698

**Published:** 2023-11-27

**Authors:** Hajar AlQahtani, Bashayer AlShehri, Nada AlSuhaibany, Shuroug Alowais, Mariam Alsulimani, Majd Alyaqoub, Lama Alkhathran, Ameerah Alghanim, Hessa AlQahtani, Mohsen AlZahrani, Mohammed Bosaeed

**Affiliations:** National Guard Health Affairs, RIYADH, Ar Riyad, Saudi Arabia; NGHA, Riyadh, Ar Riyad, Saudi Arabia; King Saud Bin Abdulaziz University for Health Science, riyadh, Ar Riyad, Saudi Arabia; King Saud University of Health Science, riaydh, Ar Riyad, Saudi Arabia; King Saud Bin Abdulaziz University for Health Science, riyadh, Ar Riyad, Saudi Arabia; King Saud Bin Abdulaziz University for Health Science, riyadh, Ar Riyad, Saudi Arabia; King Saud Bin Abdulaziz University for Health Science, riyadh, Ar Riyad, Saudi Arabia; King Saud Bin Abdulaziz University for Health Science, riyadh, Ar Riyad, Saudi Arabia; King Saud Bin Abdulaziz University for Health Science, riyadh, Ar Riyad, Saudi Arabia; National Guard Health Affairs, RIYADH, Ar Riyad, Saudi Arabia; National Guard Health Affairs, RIYADH, Ar Riyad, Saudi Arabia

## Abstract

**Background:**

The suppression of immune system in transplant patients increases the risk of Cytomegalovirus reactivation, which leads to higher rates of morbidity and mortality. Many risk factors have been identified to be associated with increased risk of CMV reactivation including mismatched human leukocyte antigen (HLA) transplant and myeloablative conditioning.

**Methods:**

All patients =/ > 14 years that underwent allo-SCT from April 2014 to April 2022 with complete medical records were included. Data collected include CMV serostatus, CMV viral load, time to CMV reactivation, conditioning regimen, use of in-vivo T-cell depletion for graft vs. host disease (GvHD)prophylaxis, and GvHD prophylaxis medications if used

**Results:**

We included 404 patients with an average age of 29 years and 42% of female sex. Patients were divided into two different groups based on underlying disease for which All-SCT was indicated.183 patients (Group 1) underwent Allo-SCT for underlying hematological malignancy mainly acute myeloid leukemia (N= 64) and acute lymphoblastic leukemia (N=73) and remaining patients distributed between CML, Hodgkin and non- Hodgkin lymphomas. Others (N = 221) had sickle cell anemia and a plastic anemia. More than 75% of patients received myeloablative conditioning regimen in Group1 compared to Group2 were most patients received non-myeloablative regimen. Most patients received matched – related human leukocyte antigen (HLA). CMV seropositive recipients constitute 95% of all patients; 89% of patients experienced CMV reactivation within 13 days post SCT and had an average initial viral load of 51 IU during reactivation. 220 patients (62%) were able to clear CMV spontaneously within a median of 50 days post reactivation. 90 day non-relapse mortality and 1 years overall survival were 2.7% and versus 1.4% and 80% versus 94% in Group 1 and Group 2 respectively.

**Conclusion:**

Our population is considered at higher risk of CMV reactivation due to the high prevalence of CMV seropositivity. Identifying factors that could contribute to CMV reactivation would help in identifying patients at highest risk and therefore, identify the best approach to manage CMV reactivation in those patients.

**Disclosures:**

**All Authors**: No reported disclosures

